# Compressed Nitrous Oxide

**Published:** 1883-01

**Authors:** Alfred Coleman


### ARTICLE V.

### *Remarks on Compressed Nitrous Oxide Gas.*

BY ALFRED COLEMAN, L. R. C. P., F. R. C. S., L. D. S , &C.

Nitrous oxide was first liquefied by Faraday, who employed the following method: Some nitrate of ammonia was placed at one end of a strong glass tube, bent near its centre at an angle, and then the other end was hermetically sealed. The end containing the salt was exposed to heat, and the gas thus generated exerted so prodigious a pressure that it became liquefied in the other extremity, which was kept cool. The pressure was ascertained to be 50 atmospheres equal to 750 lbs. on the square inch at 45° Fahr. The experiment was a very dangerous one, and the experimenter ran considerable risk, even though his face was protected with a wire mask and his hands with thick gloves.

Faraday afterwards accomplished the same object by direct pressure. He employed two condensing syringes fixed to a table, the first having a piston of an inch in diameter, and the second a piston of only half an inch in diameter, and these were so associated by a connecting pipe that the first pump forced the gas into and through the valves of the second, and then the second could be employed to throw forward this gas, already condensed, to ten or twenty atmospheres into its final recipient, the condensing tube, at a much higher pressure.* This is much the same process as is now adopted for liquefying the gas, and the apparatus as employed by Messrs. Coxeter was exhibited at the interesting lecture delivered by Mr. Braine before the Odontolog-

* 'Elements of Chemistry.' By Thomas Graham, F. R. S. Vol. i, p. 71.

ical Society, May 6th, 1872. According to a well-known law gases expend in a certain proportion for each increment of heat, but what this is for nitrous oxide has not been very accurately determined. Accordingly to Roscoe and Schorlemmer,† the pressure necessary for the liquefaction of nitrous oxide is 30 atmospheres (450 lbs.) on the square inch at 0° Cent.; consequently, as the pressure required for the same at 45° Fahr. is, according to Faraday, 50 atmospheres, this would give 20 atmospheres for 13° Fahr., or over 1½ atmospheres to each degree Fahr. Messrs. Barth & Co. inform me that they calculate an increase of 100 lbs. pressure for each 10° Fahr., or 10 lbs. (⅔ atmosphere) for each degree Fahr., or rather more than 1 atmosphere for each degree Cent., and this again corresponds more closely with Faraday's table for carbonic acid; thus at 0° Fahr. the tension of that gas is 22·84 atmospheres, and at 32° Fahr. it is 38·50 atmospheres, or about 12 atmospheres for 18° Cent.; nitrous oxide being less compressible would show a higher rate, and it is, I think, probable that Roscoe and Schorlemmer mean 30 atmospheres at 0° Fahr. about—18° Cent., and not at 0° Cent. The matter is one of interest, and could be readily worked out by those who liquefy nitrous oxide on a large scale.

The bottles constructed for containing the liquid nitrous oxide, at least those supplied by the Messrs. Barth and Messrs. Coxeter,* are, I am informed, tested by the maker up to a pressure of 4400 lbs. to a square inch; they could probably resist a much greater strain, but for all practical

---

† 'Treatise on Chemistry,' vol. i, p. 417.

* I have recently seen some bottles not supplied by the above firms, which, in addition to having flat bottoms instead of dome-shaped ones—an element of weakness—have apparently no neck welded into the upper end of the bottle into which the valves are screwed, as in the bottles supplied by the above-named firms, but the valves have the appearance of being soldered into a hole made in the flat top of the bottle; or if screwed at all it must be into only a comparatively thin surface of metal and the valves would be liable to break off from a heavy blow or fall, and, perhaps, with very disastrous consequences.

purposes we must reckon their resistance at that. At a temperature of about 50° Fahr. Messrs. Barth and Co. found that directly some of the gas became liquefied a pressure-guage indicated 900 lbs. on the square inch, but this pressure was not increased when the vessel was completely filled, and which would be conformable with known laws.

In calculating the increased pressure for increased temperature we may, I think, fairly take the simple calculation of Messrs. Barth & Co., viz. 10 lbs. for each degree Fahr., and starting with Faraday's statement that liquid gas exerts a pressure of 50″ atmosphere equal to 750 lbs. at 45° Fahr. we should if the gas were raised to 212° Fahr., the boiling point of water, have a pressure on the bottles of 2420 lbs. to the square inch, thus leaving in the bottle still one-half of its capacity of resisting power. This calculation, if it be correct, is very assuring, as it shows that gas bottles must be perfectly safe for all other than extraordinary temperatures.

In the case of the bottles employed for the compressed—not liquefied—gas the conditions are very different. The bottle which caused the melancholy accident detailed in the last number was a large iron bottle calculated to hold forty-five gallons of gas compressed at 25 atmospheres equal to 375 lbs. on the square inch. Now the contents of such a bottle, which is tested to only 1000 lbs. to the square inch, would if heated up to the boiling point of water, exert a pressure of probably over 1000 lbs. on the square inch, and if so, would undoubtedly burst. Had the bottle been one for liquid gas it is more than probable that it would have attained the melting point of the solder without bursting. There can be no doubt, but that the bottles for compressed gas should be at once and forever discarded; they might even burst if left in the rays of a tropical sun.

Still assuring though the calculations may be regarding the safety of the bottles for liquid gas, there are nevertheless still questions of some moment to those who habitually employ them. The first may be, does the liquid gas in any manner act upon the iron of the bottles? We should apprehend it would not, and I am informed that it has no corrosive action upon them whatever. In the second place, it may be asked are the bottles re-tested on each occasion of their being re-filled, and can we rely on their retaining the original pressure test? When liquid nitrous oxide was first introduced, I insisted on our having some guarantee of the resisting power of each bottle when sent out full of gas. I urged the matter strongly on the attention of the Odontological Society, and was, I recollect, considered fussy—disagreeably so—in the matter. To the Messrs. Coxeter I suggested that each bottle before being sent out should when filled be immersed for some time in boiling water; they expressed their willinguess to adopt this test, if desired, but pointed out the undesirableness of continually exposing the bottles to a severe strain as likely to weaken them. I think, however we might ask that each bottle before being refilled should be tested by hydraulic pressure, a process far less likely to injure them than an elastic pressure, and very easily carried out to say 3000 on the square inch.

But the most important question to my mind is what would be the condition of an operating room, for instance, containing gas bottles if it took fire; who would dare enter such a room to extinguish the flames, and it they did what frightful risk would they not run? That gas bottles have survived such a catastrophe is no argument that they should always do so. To obviate this danger I have suggested, and the idea has occurred to others, that each bottle should have a small hole drilled from its exterior near the bottom diagonally upwards into its interior, and that this should be filled up with a metal melting at or below 400° Fahr. from its direction the pressure would be exerted on it to only the smallest degree, whilst in the case of fire the bottle would discharge itself with, at all events, the minimum of dangerous effects, a little hot spelter being scattered about.—*British Journal of Dental Science.*

